# Supplementary material for: Identification of photoperiod-induced specific miRNAs in the adrenal glands of Sunite sheep (Ovis aries)
Source: Front Vet Sci. 2022 Jul 22;9:888207. doi: 10.3389/fvets.2022.888207 (PMC9354845; doi:10.3389/fvets.2022.888207)
Supplement: Supplementary file 1 [file Data_Sheet_1.ZIP › Supplementary file/miRDeep2 file.zip!/miRDeep2 file/Novel_Structure/SPLP42A3/Novel_250.pdf]

Provisional ID	:	NC_019474.2_28191
Score total	:	2
Score for star read(s)	:	-1.3
Score for read counts	:	0
Score for mfe	:	1.7
Score for randfold	:	1.6
Score for cons. seed	:	
Total read count	:	391
Mature read count	:	391
Loop read count	:	0
Star read count	:	0

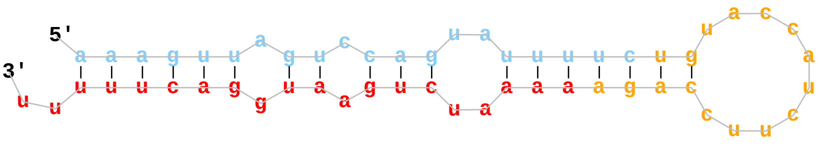

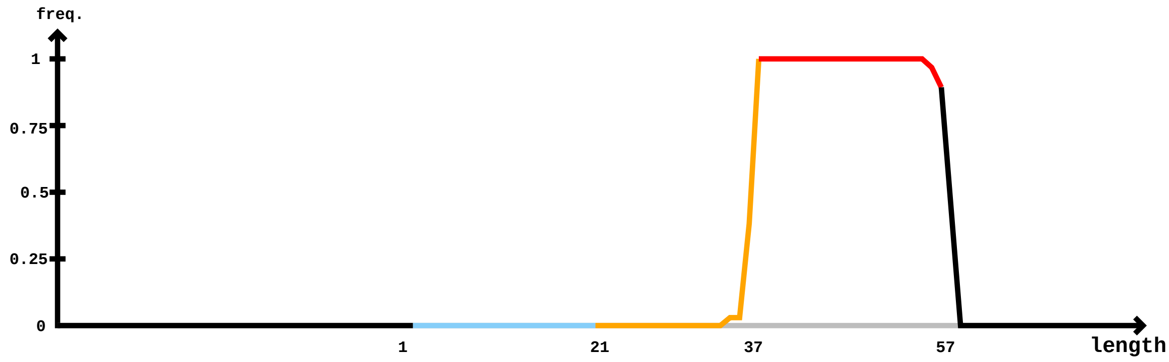

Star Mature

```
5'- ccugugauacuugugugaaauauuggauuggccaaaaaguuaguccaguauuuucuguaccaucuuccagaaaaaucugaauggacuuuuuggccaauccauauauauuug   -3'    exp
    ................((((((((((((((((((((((.((.(((..(((((((.........)))))))..))).)).))))))))))))))))...)))))).    reads    mm    sample
    ....................................................................agaaaaaucugaaugAacuuu......................    1    1    seq
    ....................................................................agaaaaaucugaaugAacuuuu.....................    3    1    seq
    ....................................................................agaaaaaucugaaugAacuuuuu....................    8    1    seq
    ......................................................................aaaaaucugaaugAacuuu......................    12    1    seq
    ......................................................................aaaaaucugaaugAacuuuu.....................    26    1    seq
    ......................................................................aaaaaucugaaugAacuuuuu....................    78    1    seq
    ......................................................................aaaaaucugaauggacuuuuu....................    1    0    seq
    ......................................................................aaaaaucugaaugAacuuuuug...................    24    1    seq
    ......................................................................aaaaaucugaauggacuuuuug...................    1    0    seq
    .......................................................................aaaaCcugaauggacuuuu.....................    1    1    seq
    .......................................................................aaaaucugaauggacuuuuu....................    1    0    seq
    .......................................................................aaaaucugaaugAacuuuuu....................    247    1    seq
```
